# Harmful Impact of Tobacco Smoking and Alcohol Consumption on the Atrial Myocardium

**DOI:** 10.3390/cells11162576

**Published:** 2022-08-18

**Authors:** Amelie H. Ohlrogge, Lars Frost, Renate B. Schnabel

**Affiliations:** 1 Department of Cardiology, University Heart and Vascular Centre Hamburg, 20246 Hamburg, Germany; 2 German Centre for Cardiovascular Research (DZHK), Partner Site Hamburg/Kiel/Lübeck, 20246 Hamburg, Germany; 3 Diagnostic Centre, University Clinic for Development of Innovative Patient Pathways, Silkeborg Regional Hospital, 8600 Silkeborg, Denmark; 4 Department of Clinical Medicine, Aarhus University, 8200 Aarhus, Denmark

**Keywords:** atria, atrial myocardium, atrial cardiomyopathy, tobacco, cigarettes, smoking, alcohol, drinking

## Abstract

Tobacco smoking and alcohol consumption are widespread exposures that are legal and socially accepted in many societies. Both have been widely recognized as important risk factors for diseases in all vital organ systems including cardiovascular diseases, and with clinical manifestations that are associated with atrial dysfunction, so-called atrial cardiomyopathy, especially atrial fibrillation and stroke. The pathogenesis of atrial cardiomyopathy, atrial fibrillation, and stroke in context with smoking and alcohol consumption is complex and multifactorial, involving pathophysiological mechanisms, environmental, and societal aspects. This narrative review summarizes the current literature regarding alterations in the atrial myocardium that is associated with smoking and alcohol.

## 1. Introduction

The EHRA/HRS/APHRS/SOLAECE expert consensus group defines atrial cardiomyopathy as “any complex of structural, architectural, contractile, or electrophysiological changes affecting the atria with the potential to produce clinically-relevant manifestations” [1].

The most commonly used classification for histopathological structural changes is the EHRAS classification, distinguishing between “(I) principal cardiomyocyte changes, (II) principally fibrotic changes, (III) combined cardiomyocyte-pathology/fibrosis, (IV) primarily non-collagen infiltration (with or without cardiomyocyte changes)” [1].

Architectural and contractile changes can be evaluated with imaging, such as echocardiography or in magnetic resonance imaging (MRI) or computer tomography (CT). While echocardiography is more easily available and can show alterations in dimension and contractility, the MRI is superior to evaluate myocardial changes such as fibrosis. The Utah classification is commonly used to classify atrial fibrosis that is visualized by late gadolinium enhancement (LGE) in MRI into stages I–IV [2], although not without limitations.

Clinically, electrophysiological changes are the most commonly evaluated using an electrocardiogram (ECG), however, electroanatomic mapping that is generated in invasive electrophysiological examinations provides more detailed information on the quality, quantity, and localization of arrhythmogenic substrate. Experimentally, laboratory testing in isolated human or animal myocytes can provide insights into the functions of the various ion channels and the resulting action potential.

Clinically relevant manifestations include atrial fibrillation (AF), stroke, and systemic thromboembolism. Stroke is a heterogenous disease which can be subdivided into haemorrhagic and ischaemic stroke. While the first makes up for about 20% of all strokes and is mostly attributable to large-vessel disease with the major risk factor being hypertension, the latter represents the majority (about 80%) of strokes and can be further subdivided. The most important categories of ischemic stroke are cardioembolic, large vessel, and microangiopathic stroke. Atrial cardiomyopathy is typically associated with cardioembolic stroke, though there is an overlap of risk factors with other types of stroke as well.

No specific biomarker to predict atrial cardiomyopathy has been identified. The strongest markers for the prediction of AF are natriuretic peptides such as atrial natriuretic peptide and the clinically more common B-type natriuretic peptide or its precursor fragment, N-terminal pro B-type natriuretic peptide (NT-proBNP). Other biomarkers of cardiac damage, such as troponin or inflammatory markers, such as C-reactive protein (CRP) have also been identified. Further biomarkers are under investigation. A more comprehensive overview can be found in several reviews [3,4,5].

Exposure to smoking and alcohol is almost universal and legal in most countries in the world and often deemed “socially acceptable” in contrast to most other drugs and narcotics. However, tobacco and alcohol have been recognized as serious public health issues for decades. According to the World Health Organization, in 2016, 43% of the adult world population had consumed alcohol within the previous 12 months with high regional variations, another 12.5% are classified as former drinkers [6]. About 20% of the global adult population were current smokers in 2015 [7]. Ethical principles forbid the execution of large randomized controlled trials in humans to directly examine the effects of chronic smoking and alcohol consumption, but randomized withdrawal can be studied. Thus, predominantly observational data are available, and these results need to be regarded cautiously and with possible biases and confounders in mind. With regards to atrial cardiomyopathy, these uncertainties are particularly relevant for clinical outcomes. Smoking and alcohol predispose people to cancer, and to several cardiovascular risk factors (CVRF) and diseases, such as obesity, hypertension, diabetes, atherosclerosis, and heart failure which in themselves increase the risk for adverse events such as AF or stroke. Reducing these risk factors in patients with AF, including alcohol and smoking but also obesity, diabetes, and hypertension reduces AF recurrence, burden, and complications [8,9,10,11]. It is difficult to determine the share that atrial cardiomyopathy contributes these outcomes in comparison to other risk factors.

**Figure 1:**
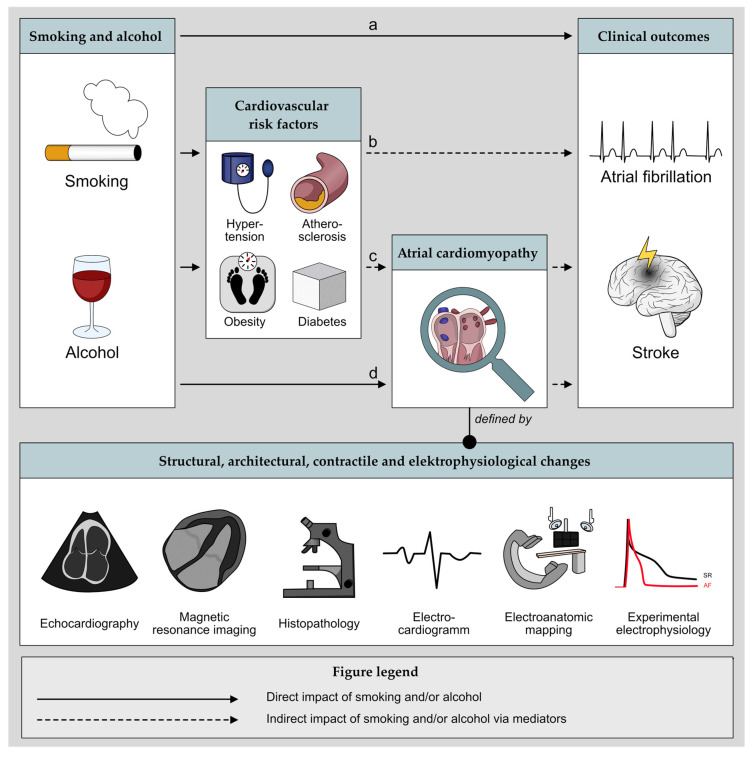
Schematic illustration of the potential interactions between smoking and alcohol cardiovascular risk factors, atrial cardiomyopathy, and clinical outcomes. Hypothetically, smoking and alcohol might (**a**) directly contribute to these clinical events without the involvement of the atria or CVRF via pathways that are not yet understood; (**b**) the CVRF might contribute to the clinical outcomes without involvement of the atria; (**c**) the conditions such as hypertension might contribute to atrial cardiomyopathy as a mediator and ultimately lead to clinical outcomes; (**d**) directly affect atrial cardiomyopathy and thus facilitating clinical outcomes.

For a schematic illustration of the potential interactions between smoking and alcohol, cardiovascular risk factors, mediators, atrial cardiomyopathy, and clinical outcomes see Figure 1.

The objective of this review is to summarize the current scientific literature examining the impact of tobacco smoking and alcohol on the atrial myocardium.

## 2. Materials and Methods

We searched the PubMed database for the terms “smoking”, “cigarettes”, “tobacco”, “alcohol”, combined with “atrial”, or “atrium” and the respective chapter search term: “echocardiography”, “MRI”, “CT”, “fibrosis”, “oxidative stress”, “remodeling”, “apoptosis”, “ECG”, “electrophysiology”, “substrate”, “ion channels”, “atrial fibrillation”, “systemic thromboembolism”, and “stroke”. The search included a maximal look back period in PubMed, and the literature was retrieved in the period from 4 April 2022 until 10 June 2022. Due to the numerous combinations of search terms and the extensive amount of literature in this field, a narrative approach was chosen for this review at the awareness of potential limitations and biases.

## 3. Atrial Myocardium and Tobacco Smoking

The most pharmacologically relevant and addictive component of tobacco is nicotine, which is also available in electronic cigarettes (EC) as well as substitute products, such as plasters and chewing gums, that are designed to assist smoking cessation. However, almost 9600 different chemical components have been identified in total in tobacco smoke besides nicotine [12]. Many of these are recognized to be harmful, for example carbon monoxide and oxidants. The proportion to which nicotine and the other components contribute to the overall harmful effects of smoking is not yet fully resolved.

Cotinine is a metabolite of nicotine, and serum cotinine levels or urinary cotinine are frequently used biomarkers that indicate smoking burden [13].

**Figure 2:**
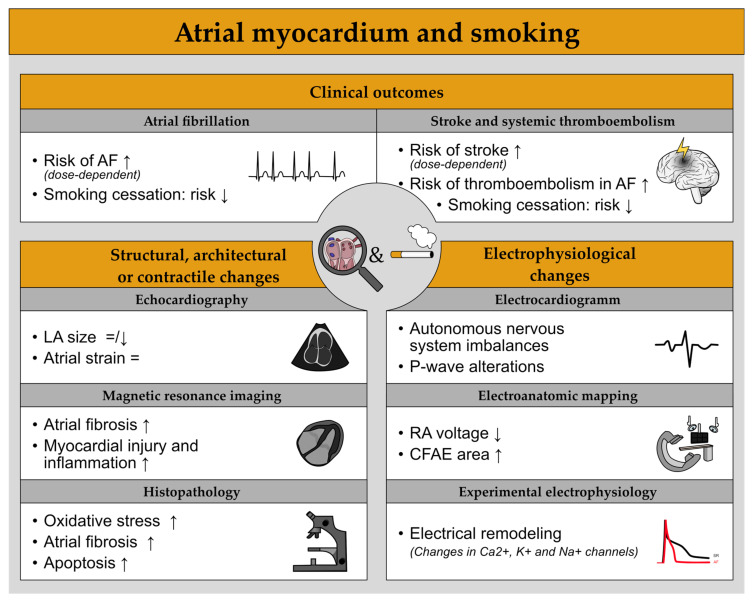
Overview of the impact of smoking on the atrial myocardium. = indicates no significant changes, ↑ an increase and ↓ a decrease of the respective aspect.

For an overview on the impact of smoking on the atrial myocardium see Figure 2.

### 3.1. Structural, Architectural, and Contractile Changes

Unlike alcohol (see Section 4.1), smoking does not seem to be associated with significantly enlarged atria. In contrast, some evidence even points towards a decreased left atrium (LA) size that is associated with smoking. The reasons for a reduction in the LA size that was observed in some studies are unexplained. A lower BMI in smokers could be considered as a cause of a smaller atrium, because of the correlation between BMI and left atrial size. A direct effect of smoking on the atrium can also be considered, but the current evidence suggests a non-significant reduction of atrial strain or increase in fibrosis by exposure to smoking, although data are limited and conflicting.

#### 3.1.1. Echocardiography

##### Atrial Size

In 3581 participants of the Framingham study, there was a significant association between smoking and LA size. A total of 16.8% of participants with a left atrial size in the highest tertile were smokers compared to 24.7% in the second and 29.0% in the first tertile of LA size [14]. In 2804 American Indian participants of the Strong Heart Study, 25.7% of the participants with an enlarged left atrium were smokers compared to 32.7% with normal left atrial size [15]. Likewise, among 1886 participants of the Atherosclerosis Risk in Communities (ARIC) study, fewer participants were current smokers in the highest quintile of LA size compared to the lower four quintiles, although without reaching statistical significance [16]. In contrast, in 90 patients with an enlarged left atria, smoking was more common than amongst 429 patients with normal sized atria [17]. Left atrial dimensions were found not to be associated with smoking in 2903 participants in the CARDIA study [18].

##### Atrial Strain

In a study of 80 healthy smokers and 70 healthy non-smokers, smokers had significantly lower atrial reservoir and conduit strains for both the right and left atrium [19]. No statistically significant difference was seen between 119 smokers and 266 non-smokers in left atrial reservoir strain [20] or between 121 current, 121 former, and 125 never smokers for peak atrial longitudinal strain [21].

#### 3.1.2. MRI and CT

##### Atrial Size

Smoking was associated with a smaller LA area index in the population-based sample for the DANCAVAS trial with 10,902 men without AF in non-contrast CT measurements [22]. Likewise, among 3945 participants from the community-based Heinz Nixdorf Recall Study, current smokers had a significantly reduced LA size [23].

In the Multi-Ethnic Study of Atherosclerosis (MESA) with 2576 participants, smoking was not associated with LA volume index, though there was a significant association between smoking and non-indexed LA volume [24].

##### Atrial Fibrosis and Scarring

Imaging-based data in relation to atrial and myocardial fibrosis by exposure to smoking are conflicting. In the MESA study, 143 out of 2839 participants had myocardial scar in an MRI. Smoking was more common in the scar group compared to the matched control group. Scarring was associated with higher LA volume and reduced LA ejection fraction and LA strain [25]. In 68 patients with acute myocarditis, a strong correlation between smoking and LGE extent was observed [26].

No association was found between the smoking status and LA fibrosis by Utah Stages in cardiac MRI in 308 patients that were undergoing ablation of AF, although smoking was associated with a higher arrythmia recurrence rate after AF ablation [27]. Also, no significant association between smoking and Utah stages for LA fibrosis were found in another MRI study, although with 81 participants this study was comparatively small [28]. A study with 88 subjects found no significant association between late gadolinium enhancement (LGE) in left atrium and smoking [29].

#### 3.1.3. Histopathology

##### Fibrosis

**Figure 3:**
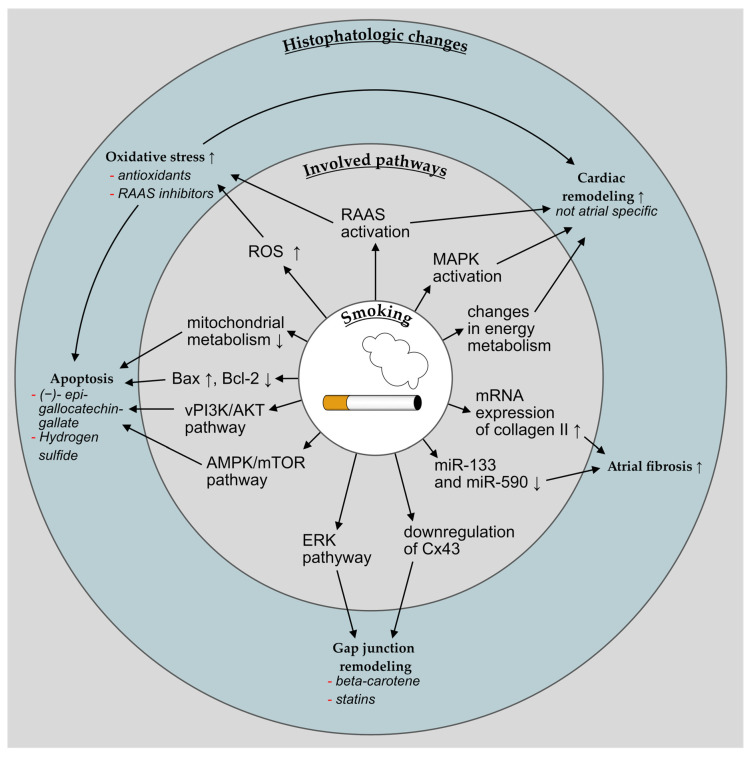
Simplified overview over the histopathologic changes in the atrium that are associated with smoking and the involved pathways. ↑ indicates an increase and ↓ a decrease of the respective aspect, —an attenuation of a certain observation.

The literature provides information of the pathways of fibrogenic mechanisms in other organs, in which nicotine-induced fibrosis occurs, such as the lungs or vessels. Here, amongst others, growth factors, inflammation, oxidant balance, and fibroblast activation appear to play a role in the genesis of fibrosis and atherosclerosis [30,31,32]. The molecular pathways potentially differ from those that are involved in atrial fibrosis, and the extent of overlap between cardiac and non-cardiac nicotine-induced pro-fibrotic mechanisms currently is unclear. For a simplified overview see Figure 3. In 95 patients that were undergoing coronary artery bypass grafting (CABG), the right atrial appendages were obtained during surgery and further examined. Pack years were significantly associated with atrial fibrosis in smokers, and atrial fibrosis was associated with a significantly increased risk of postoperative AF. In atrial tissue slices, messenger RNA (mRNA) expression of collagen II was significantly induced by the presence of a nicotine base in a up to 10-fold concentration-dependent manner [33].

In a canine model in which atrial fibroblasts from healthy dogs were treated with nicotine, increased AF vulnerability and stimulated collagen production and atrial fibrosis were observed in vitro and in vivo. In further examinations, nicotine-induced downregulation of microRNAs (miR), specifically miR-133 and miR-590 and thus upregulated the transforming growth factor-β1 (TGF-β1) and TGF-β receptor type II (TGF-βRII) [34].

There is plenty of evidence on the harmful effect of smoking and of nicotine in relation to the impact on oxidative stress in different organ systems [35,36,37,38]. Anti-oxidative substances have been shown to attenuate or reverse these effects, at least partly [39,40,41]. This oxidative stress and other factors lead to cardiac remodeling. However, many of the following studies describe ventricular, not atrial, remodeling. Differences between atrial and ventricular remodeling have been observed for some mechanisms [42,43]. Other general mechanisms that are responsible for ventricular remodeling, such as oxidative stress and activation of the renin-angiotensin-aldosterone-system (RAAS) have been described for atrial as well as ventricular remodeling [44,45]. Some studies examining the effect of smoking on cardiac remodeling describe changes in the atrium as well [46,47], so at least partial commonalities between atrial and ventricular remodeling can be assumed.

In rats, nicotine administration worsened ischaemia-reperfusion injury and increased the mitochondrial production of reactive oxygen species (ROS) [48]. In a follow-up study, it was found that this effect could be reduced by the administration of an angiotensin II type I receptor antagonist [49]. Also in rats, nicotine administration for 28 days induced higher ROS levels, fibrosis hypertrophy of cardiomyocytes, and inflammation. This effect could be reduced by the addition of mitoTEMPO, targeting mitochondrial ROS, and resveratrol as a sirtuin activator [50]. Similar effects of cigarette smoke on oxidative stress and cardiac remodeling have been shown in mice [51,52].

In mice, exposure to cigarette smoke induced multiple damages to the myocardium, including reduced contractile function, Ca^2+^ mishandling, fibrosis, apoptosis, and mitochondrial damage. Cardiac-specific overexpression of metallothionein was protective against these effects [53]. Exposure to tobacco smoke induced cardiac remodeling via oxidative stress in rats in several studies [54,55]. A variety of antioxidants and antagonists of the RAAS were demonstrated to attenuate this [46,47,56,57,58,59,60]. An increase of glucose metabolism was found in the early phase of cardiac remodeling after tobacco smoke exposure, with an increase in glycolytic pathways [58] and insulin resistance [61]. Other changes in myocardial energy metabolism of rats that was induced by cigarette smoke, such as lipotoxity, have been reported as well [62].

Cigarette smoke exposure affected the myocardial mitochondrial metabolism and induced apoptosis in rats by the activation of caspase-3, cytochrome c release and regulation of pro- and anti-apoptotic molecules such as Bax and Bcl-2. The antioxidant (−)-epigallocatechin-gallate reversed these effects [63]. Hydrogen sulfide (H2S) attenuated cardiac damage that was induced by cigarette smoking in rats by reducing apoptosis and autophagy and also antioxidant effects via the PI3K/Akt signaling and AMPK/mTOR signaling pathways [64,65]. Another study reported a MAPK activation that was induced by cigarette smoke in rats and was associated with ventricular remodeling [66].

Cell membranes were affected in their function and integrity by cigarette smoke in human cardiac stem cells via oxidative stress and ERK-signaling [67]. In rats and human umbilical vein endothelial cells, cigarette smoke exposure resulted in remodeling of cardiac gap junction with the downregulation of connexin 43 (Cx43) and changes in phosphorylation [68,69,70]. These effects could be attenuated by beta-carotene as an antioxidant and statins via an unknown pathway [68,71]. Overall, most of these processes have been demonstrated in ventricular myocardium and atrial-specific studies are missing and only analogy conclusions that similar pathophysiological pathways are likely to be involved in atrial myocardium can be drawn.

### 3.2. Electrophysiological Changes

#### 3.2.1. ECG

Generally, smoking can lead to several ECG abnormalities [72,73]. Some of these are most likely caused by the increase in CVRF and myocardial ischaemia, such as signs of left ventricular hypertrophy and ST-segment alterations. Others are associated with atrial changes, such as deep terminal negativity of the P-wave in V1 (DTNPV1) [74,75,76] or reduced heart rate variability (HRV), a marker of the balance in the autonomous nervous system [77,78].

##### Autonomous Nervous System

In a cross-sectional study with 1218 non-smokers that were aged 50 or older with 24-h ECG-recordings, exposure to environmental tobacco smoke for more than 2 h a day was associated with ECG changes that indicate disturbances in the autonomous nervous system, such as a lower total power and frequency power, a lower low/high frequency ratio, and ultralow frequency power of HRV compared to subjects that were not exposed to tobacco smoke [79]. Similar alterations were reversible after 15–25 years of smoke cessation, depending on the intensity of former smoking, in 1481 participants over 50 years of age [80]. Several smaller studies have confirmed a reduction in the HRV by smoking [81,82,83,84]. Correspondingly, second-hand exposure to smoke reduced heart rate variability in mice [85].

In a study among 31 male smokers and 15 healthy non-smokers the acute influence of smoking on the ECG was examined. It was observed that the heart rate increased, the occurrence of ectopic beats increased, and HRV index decreased [86].

##### P-Wave Alterations

In a study of 8146 individuals that were aged 40 and above without AF, current smokers were statistically significantly more likely to present a DTNPV1 in their ECG, which was associated with an increased risk of death [87]. Also, in 4507 patients without AF, abnormal DTNPV1 was associated with elevated serum cotinine levels [88].

#### 3.2.2. Electroanatomic Mapping

In 88 patients that were undergoing PVI for paroxysmal AF, the mean voltage and total activation time were obtained during the procedure. The right atrial mean voltage was found to be lower in smokers compared to never-smokers, with a significant dose-dependent effect. Also, the total activation time of the right atrium was longer. No significant differences were observed for the left atrium [89].

In a study including 120 AF patients and 120 controls, smoking showed a moderate but statistically significant positive correlation to the total complex fractionated atrial electrogram (CFAE) area [90].

#### 3.2.3. Experimental Electrophysiology

Nicotine affects atrial inward rectifier potassium, acetylcholine-sensitive current I K(Ach) in isolated rat atrial myocytes [91]. It also blocks transient outward K^+^ current (Ito), delayed rectifier K^+^ currents (IKr), and inward rectifier K^+^ currents (IK1) in canine myocytes [92,93]. Ionic currents (ICa) were blocked by high nicotine doses in guinea pig ventricular cardiomyocytes and rabbit sinoatrial nodal cells [94,95]. In rats, the inducibility of atrial tachycardia and AF was age-dependent [96].

Additionally, nicotine causes a negative inotropic and chronotropic effect in the right and left atrium of rats [97].

Carbon monoxide (CO) caused a reduced action potential duration in isolated rat atrial and ventricular myocardial cells as well as a decrease in contractile force [98].

### 3.3. Clinical Outcomes

#### 3.3.1. Atrial Fibrillation

Smoking has been found to increase the risk of incident AF in numerous clinical studies [99,100,101,102,103,104,105] and in one mendelian randomization study [106]. Some studies report a dose-dependency between risk increase and the amount and/or duration of smoking [100,103,105], whereas other studies could not observe a dose-dependency [99,102]. The examination of this effect in meta-analyses is difficult due to different categorizations and cut-offs across studies. One meta-analysis has performed such an analysis and found a clear dose-dependency [107]. Second-hand smoking exposure during pregnancy and in childhood was associated with incident AF later in life [108,109]. Nonetheless, smoking did not increase the lifetime risk of AF in the Framingham cohort, due to earlier all-cause mortality among smokers [110]. A more comprehensive summary can be found in various meta-analyses and reviews [107,111,112].

##### Smoking Cessation

Smoking cessation after AF diagnosis appears to reduce AF recurrence risk [102] as well as the recurrence risk after pulmonary vein isolation (PVI) [113,114,115] and cardioversion, at least for women [116]. Conflicting data exist regarding the AF risk of former smokers, with some studies showing a reduced AF risk for former smokers compared to current smokers [103,117], whereas other studies could not confirm this finding [98].

##### Non-Cigarette Nicotine Consumption

Due to their relatively new introduction to the market, the impact of EC smoking on AF is not yet well understood. While smoking of EC can reduce the consumption of cigarettes or help with quitting tobacco smoking [118,119,120], there is no proof that this reduces the occurrence of AF. On the contrary, the flavors that ore often used in ECs affect cardiac electrophysiology in mice [121]. Some case reports show an association of EC smoking with incident AF [122,123]. ECs have been compared to the consumption of snus, a widespread Scandinavian smokeless form of tobacco, which did not show an increased risk of AF in a large Swedish cohort study [124] but increased the risk of heart failure [125]. Further research is necessary to understand the health implications that are posed by electronic cigarettes. Some case reports also describe an association between nicotine replacement therapy and AF, suggesting a link between nicotine and AF regardless of the form of application [126,127,128]. A series of case reports show that not only cigarette smoking, but also marijuana smoking is associated with incidental AF [129].

Another alternative to cigarettes is heated tobacco, although we could not find any relevant evidence on the impact of heated tobacco on the atrial myocardium.

#### 3.3.2. Stroke and Thromboembolism

Several meta-analyses have thoroughly summarized dozens of studies examining the dose-dependent risk increase of smoking for the occurrence of stroke [130,131,132,133].

Smoking cessation reduced the risk of stroke without a previous AF diagnosis [134,135], after an AF diagnosis [136,137,138,139], and of recurrent stroke [140,141,142]. In two large US health surveys, it has been observed that contrary to the general population, the prevalence of current smoking has not decreased among stroke survivors [143]. It was found that the majority of smokers attempts to quit after a stroke, although only a minority succeed [144]. In contrast, a meta-analysis of 25 prospective studies reported a smoking cessation rate of slightly above 50% after stroke [142].

##### Risk of Stroke and Thromboembolism in AF

In patients with previously diagnosed AF, smoking was associated with an increased risk of thromboembolism or death among participants in the large Danish Diet, Cancer, and Health study, even when adjusted for stroke risk factors [145] as well as in 1222 participants from the ARIC and 756 participants from the Cardiovascular Health Study (CHS) [146]. In 2102, non-valvular AF patients from the Shinken database, current smoking was associated with increased risk of ischaemic stroke [147]. Similarly, smoking increased the risk of intracranial bleeding, all-cause mortality, and death from stroke in 426 patients with AF [137]. On the other hand, a meta-analysis from 2016 could not confirm an association between smoking and an increased risk of stroke or thromboembolism in patients with AF, but instead reported an increased risk of all-cause and cardiovascular death for smokers [148]. Besides the numerous changes to the atrium that are listed above, there are several systemic changes that are associated with smoking, such as increased systemic atherosclerosis, and increased platelet aggregation and adhesion that are contributors to an increased stroke risk [149].

## 4. Atrial Myocardium and Alcohol

**Figure 4:**
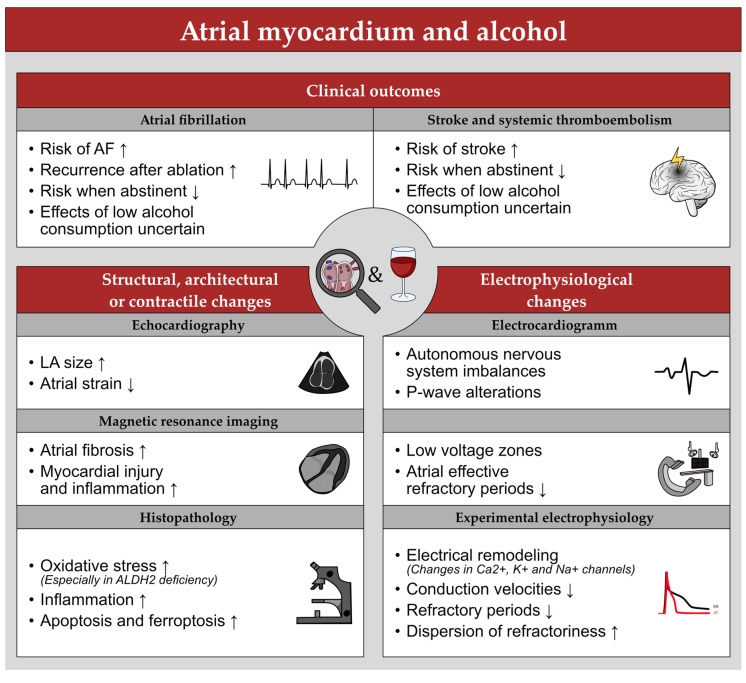
Overview of the impact of alcohol on the atrial myocardium. = indicates no significant changes, ↑ an increase, and ↓ a decrease of the respective aspect.

For an overview on the impact of alcohol on the atrial myocardium see Figure 4.

### 4.1. Structural, Architectural, and Contractile Changes

Unlike for smoking (see Section 3.1), there is strong evidence of an association between alcohol consumption and LA enlargement. There is also some evidence for a reduction of LA strain. Both seem to be related to the amount of alcohol consumption in a dose-dependent manner.

#### 4.1.1. Echocardiography

##### Atrial Size

In 5220 participants of the Framingham Heart Study, alcohol was identified as a predictor of LA enlargement and incident AF in a dose-dependent manner [150]. Similarly, in a cross-sectional study in rural China with 10,211 participants that were aged 35 and above, moderate and heavy alcohol consumption was significantly associated with the risk of LA enlargement [151]. Among 601 participants with stable coronary heart disease with echocardiographic measurements at baseline and 5 years later, alcohol use at baseline was associated with an increase in left atrial volume [152]. In 192 patients that were undergoing AF ablation, those with ethyl glucuronide in hair (hEtG) over a cut-off of 7 pg/mg had an increased LA volume [153].

##### Atrial Strain

LA strain was reduced with increasing alcohol consumption in a dose-dependent manner even for low to moderate alcohol consumption in 3946 participants [154].

Aldehyde dehydrogenase 2 (ALDH2) polymorphism (ALDH2*2) is common in Asia. In a study of 249 Asians, modest alcohol consumption was associated with an accumulation of 4-hydroxy-trans-2-nonenal (4-HNE), an ROS-generated aldehyde adduct, prolonged PR interval in the ECG, and a reduction in echocardiographic peak atrial longitudinal strain (PALS) and phasic strain rates. In individuals with ALDH2 polymorphism, an increased association between the daily alcohol intake and LA ejection fraction, PALS and phasic reservoir, and booster functions was observed [155].

Acute alcohol consumption was associated with an increased interatrial electromechanical delay that was measured by a prolongation of Pmax and Pd in tissue doppler imaging in 30 healthy men [156].

#### 4.1.2. MRI

##### Atrial Size

In 4335 participants from the UK Biobank population-based study, an association between alcohol consumption and increased left atrial volume was detected only for women. For men, alcohol consumption was associated with changes in the ventricular volume [157]. Among 160 AF patients that were undergoing cardiac MRI, self-reported regular alcohol consumption was associated with LA enlargement, reduced LA ejection fraction, and reservoir function in comparison to lifelong non-drinkers [158].

##### Myocardial Injury

In a study of 28 healthy participants, excessive alcohol consumption was associated with a significant increase in the mean myocardial T2-signal intensity one day after drinking, accompanied by pericardial effusion in three participants and also elevated high-sensitivity cardiac troponin I in 6 out of 12 examined patients, suggesting myocardial injury and inflammation [159].

#### 4.1.3. Histopathology

##### Oxidative Stress, Inflammation, and Apoptosis

**Figure 5:**
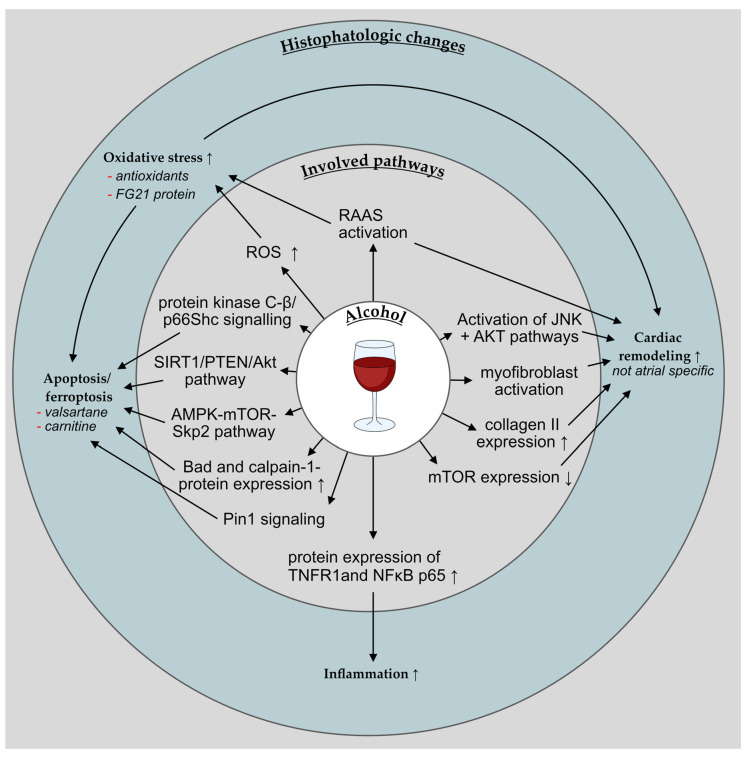
Simplified overview over the histopathologic changes in the atrium that are associated with alcohol and the involved pathways. ↑ indicates an increase and ↓ a decrease of the respective aspect, —an attenuation of a certain observation.

There is plenty of evidence on the damage that is inflicted on cardiomyocytes by alcohol via multiple inflammatory pathways and oxidative stress, which ultimately lead to apoptosis and cardiac remodeling (see Figure 5). Antioxidants and angiotensin II type 1 receptor antagonists attenuate this damage. Oxidative stress is increased in ALDH2 deficiency. As with smoking (see Section 3.1.3), many of the following experiments used ventricular cardiomyocytes. Thus, these effects should be regarded cautiously.

The mechanisms in which alcohol intervenes in cardiac cell regulation and pathways are complex and not yet entirely understood. A recent metabolomic study on alcohol-induced myocardial injury found an involvement of pathways “related to the biosynthesis of unsaturated fatty acids, vitamin digestion and absorption, oxidative phosphorylation, pentose phosphate, and purine and pyrimidine metabolism” [160]. A study using cardiomyocytes that were derived from human pluripotent stem cells that were treated with alcohol showed “increased cell death, oxidative stress, deranged Ca^2+^ handling, abnormal action potential, altered contractility, and suppressed structure development”. Proteomic profiling revealed an affection of proteins that were “involved in apoptosis, heart contraction, and extracellular collagen matrix” [161].

In mice with chronic alcohol consumption, protein expression of tumor necrosis factor (TNF)-α receptor 1 (TNFR1) and NFκB p65 was increased in cardiomyocytes. In wild-type mice, increased levels of ROS and pro-inflammatory proteins were found, but not in TNFR1-deficient mice, suggesting TNFR1-dependent mechanisms [162]. Chronic alcohol exposure in mice activated inflammatory pathways and induced myofibroblast activation and collagen II expression as well as inflammation, activation of JNK and Akt pathways, and a reduced expression of mTOR leading to cardiac remodeling [163]. In another mouse model, acute alcohol exposure led to increased apoptosis, inflammation, and mitochondrial O^2^ production in wild-type, but to a lesser extent in CD74 knockout mice. Further, autophagy was upregulated, possibly through an AMPK-mTOR-Skp2 pathway [164]. Alcohol accelerated the degradation of cardiac Fas-activated serine/threonine kinase (FASTK) mRNA. The deletion of FASTK worsened alcohol damage, whereas cardiac overexpression of FASTK was protective against alcoholic cardiomyopathy [165]. In cardiomyocytes that were isolated from rats and treated with alcohol, an increase of ROS was found leading to apoptosis and—in higher doses—to necrosis. The antioxidants vitamin E and vitamin C ameliorated oxidative stress and apoptosis [166]. Cytochrome P-450 2E1 (CYP2E1) was increased in dogs receiving chronic alcohol and was associated with markers of oxidative stress. An increased expression of pro-apoptotic Bad and calpain-1 protein was observed, this increase was inhibited by treatment with valsartan or carnitine. [167]. Likewise, in a recent study treatment with valsartan reduced alcohol-induced cardiomyocyte damage and apoptosis in human stem cell-derived cardiomyocytes. Real-time PCR analysis revealed that alcohol-activated angiotensin II and angiotensin II type 1 receptor expression resulted in increased ROS production [168]. In mouse primary cardiomyocytes, alcohol-induced cardiac apoptosis could be suppressed by siRNA-mediated knockdown of Pin1 [169]. Fibroblast growth factor 21 (FGF21)-deficient mice developed a higher degree of alcohol-induced cardiac damage, oxidative stress, and mitochondrial dysfunction than wild-type mice. In human cardiac biopsies from patients with alcoholic cardiomyopathy, a positive correlation between oxidative stress and myocardial FGF21 protein levels was observed [170]. In contrast, one study examining the effect of alcohol on rat cardiomyocytes found a protective effect of moderate alcohol doses via a reduction of proapoptotic transcription factors and genes. However, also in this study, alcohol consumption induced oxidative stress. Polyphenolic antioxidants from red wine also reduced pro-apoptotic factors [171]. In another study examining rat cardiac tissue and cardiomyocytes, low alcohol exposure reduced Akt activity, caspase 3/7 activity, and oxidative stress [172].

Apoptosis was induced by high doses of alcohol via ROS in mouse cardiomyocytes. This was regulated via protein kinase C-β/p66Shc signaling [173]. Ferroptosis, a form of non-apoptotic oxidative cell death depending on iron, was activated by frequent excessive alcohol consumption leading to cardiac remodeling with an accumulation of iron and oxidative stress reaction in the atrium and increased susceptibility to AF [174]. Empagliflozin inhibited mitochondrial apoptosis via a SIRT1/PTEN/Akt pathway in a dose-dependent manner. SIRT1 is a NAD^+^-dependent histone deacetylase which has been shown to be cardioprotective by the inhibition of pro-apoptotic molecules and antagonization of oxidative stress [175].

##### ALDH2 Deficiency

ALDH2 deficiency and subsequent elevation of 4-HNE serum levels was found in mice that were prone to stroke. Protein kinase C epsilon (PKCε) phosphorylates ALDH2 and thus induces activity of ALDH2 and showed neuroprotective effects in ALDH2-deficient mice. To verify these findings in humans, the authors analyzed a cohort of 1242 individuals. Elevated 4-HNE levels were found at the study beginning in those subjects who later developed a stroke compared to gender and age-matched controls [176]. In patients and knock-in mice with ALDH2 deficiency that was caused by the common polymorphism ALDH2*2, atrial substrate remodeling and oxidative stress was greater than in non-carriers. This mechanism is possibly transferred through the accumulation of 4-HNE. Treatment with an ALDH2-activator reduced the expression of transforming growth factor beta 1 (TGF-β1) and atrial collagen deposition [177]. In another mouse model, ALDH2*2 increased the susceptibility to AF. Multi-omics analysis revealed a reduction in the retinoic acid signals with a subsequently reduced expression of voltage-gated Na+ channels (SCN5A). Further, the dysregulation of fatty acid β-oxidation, adenosine triphosphate synthesis, and activated mitochondrial oxidative responses were observed. The latter effect could be attenuated by the administration of the anti-oxidant coenzyme Q10 [178]. ALDH2 was also found to modulate the aldosterone pathway in cardiac myocytes [179]. In reverse, transgenic overexpression of ALDH2 protected from cardiac damage in mice [180]. NADPH oxidase (NOX) activity and NOX2/glycoprotein 91phox (NOX2/gp91phox) subunit expression were increased in ALDH-2^−/−^ mice that were fed with alcohol, whereas LDH-2^−/−^/gp91phox^−/−^ mice were protected from the negative cardiac effects of alcohol. In biopsies of human patients with alcoholic cardiomyopathy, gp91phox expression was also increased. The authors suggest NOX2/gp91phox as a potential therapeutic target [181].

##### Biomarkers

Among 11,000 participants without cardiovascular disease from the ARIC study, moderate drinkers had increased NT-proBNP levels but lower high-sensitivity cardiac troponin T (hs-cTnT) [182]. Similarly, in 192 patients that were undergoing AF ablation, those with high levels of hEtG had higher levels of NT-proBNP and mid-regional fragment of pro atrial natriuretic peptide (MR-proANP) [153]. In a Russian study, 278 patients receiving treatment for alcohol problems in a narcology clinic had higher levels of hs-cTnT, NT-proBNP, and high-sensitivity C-reactive protein (hsCRP) compared to nonproblem drinkers in the general population. For harmful drinkers, compared to nonproblem drinkers from the general population only NT-proBNP was significantly elevated [183]. NT-proBNP levels increased during alcohol cessation in 55 patients with alcohol use disorder [184]. In contrast, a large study with 107,845 individuals observed only weak correlation between cardiac biomarkers NT-proBNP and high-sensitivity Troponin I (hsTnI) [185]. Acute alcohol consumption was associated with significantly elevated levels of cardiac troponin T in rats. Pre-treatment with beta-blockers lowered this increase. No elevation of cardiac troponin T could be observed for chronic alcohol consumption [186].

In six patients with a history of alcohol-induced AF, β-adrenoceptor density in lymphocytes was increased after alcohol consumption compared to six matched age-controls [187]. In 43 patients consuming moderate amounts of wine or whiskey, urinary adrenaline excretion was significantly increased [188].

### 4.2. Electrophysiological Changes

#### 4.2.1. ECG

##### Autonomous Nervous System

In 3028 participants from the MunichBREW study that was conducted at the Munich Octoberfest the autonomic tone, measured by respiratory sinus arrhythmia, was significantly reduced by acute alcohol consumption. A total of 25.9% of the participants had sinus tachycardia, which was significantly associated with breath alcohol concentration. Chronic alcohol consumption was also associated with sinus tachycardia [189].

In six patients with a history of alcohol-induced AF, low-frequency/high-frequency ratio was increased during ethanol intoxication compared to six matched age-controls, suggesting a disturbance in the autonomous nervous system balance [187]. A similar decrease in heart rate variability and an increased low-frequency/high-frequency ratio has been observed in healthy subjects [190,191,192,193,194].

##### P-Wave Alterations

The duration of the signal-averaged P-wave was increased in patients with alcohol-induced paroxysmal AF, both sober and after alcohol intake, compared to healthy controls [195]. Similarly, the P-wave duration was longer in 84 patients during acute ethanol intoxication compared to sober controls [196]. P-wave dispersion as well as maximum P-wave duration were significantly prolonged after moderate alcohol intake in 10 healthy men [197]. P-wave dispersion was also increased in 48 individuals with chronic alcohol use disorder [198].

#### 4.2.2. Electroanatomic Mapping

In a double-blinded, randomized study of 100 patients that were undergoing AF ablation, one group received intravenous alcohol infusion, titrated to 0.08% blood alcohol concentration compared to a placebo group. Exposure to alcohol significantly reduced the atrial effective refractory periods in the pulmonary veins [199].

In 122 patients with symptomatic paroxysmal AF that were undergoing PVI, daily alcohol consumption was associated with low-voltage zones in left atrial voltage mapping [200]. Similarly, among 75 patients that were undergoing AF ablation, those with regular moderate alcohol consumption had lower mean global bipolar voltages, slower conduction velocity, and a higher proportion of complex atrial potentials compared to non-drinkers. Patients with mild alcohol consumption showed less pathological findings [201].

#### 4.2.3. Experimental Electrophysiology

Several mechanisms have been reported on how alcohol consumption affects electrical functionality of the heart and increases arrhythmia susceptibility.

Binge alcohol consumption activated the stress c-Jun N-terminal kinase 2, which lead to Ca^2+^ mishandling in the sarcoplasmic reticulum via Calmodulin kinase II activation. This ultimately increased arrhythmia susceptibility in rabbit and human atria [202]. In the atria of rats with two months of alcohol consumption myofilament Ca^2+^ sensitivity as well as the effect of different inotropic drugs were reduced [203]. Also in rats that were exposed to ethanol, acetylcholine-sensitive K(+) channel Kir3.1 protein expression was significantly upregulated [204].

In pigs, ethanol infusion increased susceptibility for atrial arrhythmias that were induced by atrial stimulation [205]. Ethanol infusion over 5 days reduced atrial L-type calcium (Ica,L) and sodium (Ina) current densities in rabbits whereas the transient outward potassium (Ito), sustained (Isus), and inward rectifier (IK1) current were unchanged [206]. In rabbit single pulmonary vein, beating cardiomyocytes ethanol incubation reduced action potential duration, reduced L-type Ca^2+^ currents, and increased transient outward currents. However, beating rates and incidences of delayed afterdepolarization were similar to placebo [207].

Acute and chronic ethanol consumption increased electrical instability in rats. Conduction velocities were reduced in both atria with shortened effective refractory periods and increased dispersion of refractoriness only in the right atrium both in acute and chronic consumption. KCNQ1 and connexin40 expression were increased whereas KCNA5 expression was decreased in the right atrium in chronically ethanol exposed rats [208].

### 4.3. Clinical Outcomes

#### 4.3.1. Atrial Fibrillation

Alcohol consumption is a well-established risk factor for the occurrence of AF, for occasional, excessive drinking, and regular consumption. The term “Holiday Heart Syndrome”, describing the onset of AF after excessive drinking, was first coined in 1978 [209]. Numerous large cohort studies have since confirmed an association between alcohol consumption and incident AF [210,211,212,213,214,215,216]. Meta-analyses give a thorough overview over the extensive literature regarding the association between alcohol and AF [216,217,218,219,220,221].

##### Mild to Moderate Alcohol Consumption

While it has been well established that high levels of alcohol consumption increase the risk of AF, the evidence of the harmful effects of low to moderate amount of alcohol intake is inconclusive. Similar discussions are ongoing about the association between mild to moderate alcohol consumption and stroke (see Section 4.3.2). The definition of “moderate” alcohol consumption differs between studies and can include up to two to three standard drinks (usually about 10–14 g alcohol per standard drink with variations between countries) or 36 g alcohol per day. A moderate intake of red wine (<30 g alcohol per day for men <15 g per day for women) was not associated with AF in 6527 participants at a high cardiovascular risk from the PREDIMED trial [222]. Among 16,415 participants from the Copenhagen City Heart Study, heavy alcohol consumption (≥35 drinks per week) was associated with an increased risk of AF, at least in men, whereas moderate alcohol consumption (≤34 drinks per week) was not associated with a greater risk of AF [211]. In an analysis of 403,281 participants from the UK Biobank Study, individuals with low levels of alcohol intake (<56 g alcohol per week) had lowest AF risk compared to non-drinkers and individuals with higher alcohol intake. In further analyses, this observation was depending on the type of beverage where beer and cider were harmful even at low doses [223]. Similarly, among 10,333 individuals from the Framingham Heart study, only participants with >36 g/day of alcohol intake had a significantly increased risk of AF, whereas moderate drinking showed only a minimal and nonsignificant risk increase for the occurrence of AF [224]. In the Women’s Health Study with 34,715 healthy, middle-aged women consumption of more than two drinks per day significantly increased the risk of incident AF, whereas consumption of less than two drinks a day did not [213]. These findings are supported by a recent meta-analysis suggesting a J-shaped relationship between the amount of alcohol intake and incident AF [221].

In contrast, other studies suggest an increased AF risk even for low alcohol doses. Among 107,845 individuals from community-based cohort studies even one alcoholic drink (12 g ethanol) per day significantly increased the risk of incident AF [185]. The increased risk of incident AF even for low doses of alcohol intake is supported by two meta-analyses [216,218].

However, in the absence of randomized controlled studies, the observational nature of these data needs to be considered when interpreting these findings. The impact of light alcohol drinking might be relatively small, and thus only detected by sufficiently large studies with adequate statistical power for these small differences (see also Section 4.3.2).

##### Alcohol Abstinence

In patients with AF, evidence is emerging on the benefit of alcohol abstinence. In a randomized controlled study with 140 participants consuming 10 or more drinks a week, the abstinence from alcohol significantly reduced short-term AF recurrence as well as long-term AF burden compared to the control group that continued alcohol consumption [225]. In the ARIC study, the duration of alcohol abstinence was associated with a decrease in AF risk, with a risk reduction of approximately 20% for every abstinent decade [226].

##### AF Recurrence after Ablation

Among 1361 patients that were undergoing staged catheter ablation for paroxysmal AF, the frequency of alcohol consumption was significantly associated with increased AF recurrence after the initial ablation, however, no differences between drinkers and non-drinkers was seen after the final ablation [227]. The AF recurrence rate after radiofrequency AF ablation was higher in patients with hEtG over a cut-off of 7 pg/mg [153].

##### ALDH2 Deficiency

In a Japanese study with 656 participants, heterozygous ALDH2 deficiency (ALDH2*1/*2) carriers with habitual alcohol consumption had a greater risk of AF than ALDH2 wild-type carriers with habitual alcohol consumption. The heterogenous trait itself was not associated with an increased risk of AF. Homozygous ALDH2 deficiency (ALDH2*2/*2) was rare (4.1% of the studied population) and none of these participants consumed alcohol. These participants even had a reduced risk of AF compared to wild-type non-drinkers [228]. A Korean Mendelian randomization study with 8964 participants using the ALDH2 genotypes reported a significant relationship between alcohol consumption and AF [229].

#### 4.3.2. Stroke and Thromboembolism

Similar to AF, heavy alcohol consumption has been recognized as a risk factor for stroke in large cohort studies that were reported more than 40 years ago [230,231]. Since then, several studies have confirmed these results [232,233,234,235,236,237,238,239]. Several meta-analyses have reported reviews over the extensive literature regarding the association between alcohol and stroke and systemic thromboembolism [240,241,242,243].

##### Mild to Moderate Alcohol Consumption

Similar to the discussion around alcohol and AF (see Section 4.3.1), there are conflicting data regarding the dose-response relationship between alcohol intake and the risk of stroke. In a retrospective case-control study from 1986 with 230 stroke patients, light alcohol consumption had a relative risk of 0.5 compared to non-drinkers, whereas heavy drinker had a four-fold increased risk in comparison to non-drinkers [244]. Since then, several studies have examined this relationship. There are three meta-analyses that found an almost linear relationship between alcohol consumption and haemorrhagic stroke in men, whereas the relationship between alcohol consumption and ischaemic stroke was J-shaped with a minimal risk at one drink per day. Women had a much steeper increase in risk for ischemic and haemorrhagic stroke at higher doses of alcohol consumption than men [240,241,242]. Alcohol consumption among stroke subtypes differs, with patients with macroangiopathic stroke reporting highest, and patients with cardioembolic stroke reporting lowest rates of daily alcohol consumption in the German Stroke Data Bank [245]. A meta-analysis of 27 prospective studies found a risk reduction for low to moderate alcohol consumption for ischemic stroke, and an increased risk for all types of stroke for heavy drinking, especially for haemorrhagic stroke [243].

In a recent Korean study examining changes in alcohol drinking, sustained light drinking decreased the risk of ischaemic stroke compared to sustained non-drinking. Increasing alcohol consumption from mild to moderate increased the risk of ischaemic stroke, whereas a reduction from heavy to mild could decrease the risk of stroke [246]. A recent study with 371,463 participants from the UK Biobank found an association between light alcohol intake and healthy lifestyle factors. In both linear and nonlinear Mendelian randomization analyses, even light alcohol consumption was associated with increased cardiovascular risk (including stroke), although with large differences between the levels of intake [247]. Two other Mendelian randomization studies found similar results [248,249].

As with AF (see Section 4.3.1), the cause for the apparently protective effect of mild to moderate drinking on stroke risk is uncertain. Confounded by socio-economic characteristics, reverse causality bias, recall error, study design, and publication bias have been discussed as well as the actual benefits of alcohol consumption, such as increased HDL-cholesterol, reduced triglycerides, or favourable changes in haemostatic factors [248,250,251]. However, these effects are not generalizable to the entire population. A meta-analysis found a protective effect of light alcohol consumption for the incidence of cardiovascular disease only for those that were above 40 and with less than three comorbidities [252].

##### Alcohol Abstinence

Lifelong abstinence was significantly associated with an increased risk of stroke in a case-control study from 1993 [253]. This U-shaped relationship was also found in a prospective cohort study with 7735 middle-aged men in 1996 [254]. Alcohol consumption increased the risk of stroke in a Korean nationwide population-based cohort study with 97 869 patients with newly diagnosed AF, and abstinence of alcohol was associated with reduced risk of an ischemic stroke [255].

##### ALDH2 Deficiency

In a Taiwanese study with 914 participants, homozygous ALDH2 deficiency (ALDH2*2/*2) was found to be an independent risk factor for ischemic stroke, although only for men [256].

## 5. Limitations

While it has been established that tobacco smoking and alcohol consumption increase the risk of AF and cardiovascular diseases in general, the involved pathways and mechanisms are pleiotropic and remain only partly understood. Both tobacco smoking and alcohol consumption have a broad range of interrelating, and perhaps interacting, harmful cardiac and extra-cardiac effects that mediate negative clinical outcomes. Large cohort studies have provided plenty of data on several clinical aspects that are associated with the atrial myocardium, such as the occurrence of AF or stroke or macroscopic structural changes that are found in cardiac imaging. However, the observative nature of these studies is prone to bias. Interventional study designs involving exposure to tobacco and alcohol would ethically not be acceptable. Less evidence exists regarding other aspects within the definition of atrial cardiomyopathy, such as electrophysiological changes that are found in electroanatomic mapping and experimental studies. Most studies examining histopathologic alterations have strong limitations in their informative value that is caused by their study design. Many were performed with animal ventricular cardiomyocytes. Substances such as RAAS inhibitors or antioxidants have attenuated the damage that is inflicted both by smoking and alcohol in several experiments, but it is unknown whether such attenuation will also prevent the development of cancer. The generalizability of these in vitro findings to in vivo changes in the human atrial myocardium is difficult to determine. A more detailed understanding of the mechanisms behind atrial changes that are inflicted by tobacco smoking and alcohol consumption could potentially lead to preventive approaches, although prevention trials should also include cancer and total mortality in a composite outcome. Further research is needed to fill these gaps in knowledge.

## 6. Summary and Perspectives

Smoking and alcohol have harmful effects on the atrial myocardium, defined by changes in the cardiac structure, architecture, and contractility as well as electrophysiology and are associated with an increased risk of AF and stroke. Specific evidence of the effects of smoking and alcohol on the atrial myocardium is limited. Exposure to tobacco smoking and alcohol consumption is associated with multiple complex pathophysiological pathways involving direct and mediated mechanisms. It is, therefore, difficult to imagine that one of more harmful pathways can be effectively pharmacologically blocked, even though some substances demonstrated an attenuation of the damage that is inflicted by smoking and alcohol in vitro. While a clear dose-dependency of the harmful effect of smoking has been observed, there are conflicting data regarding the effects of low to moderate alcohol consumption on the atrium from a molecular to an epidemiologic level. The most effective public health measure to prevent exposure to tobacco and alcohol will be socioeconomic interventions, increased health literacy, and complementary market regulations.

## Abbreviations

AF—atrial fibrillation, ALDH2—Aldehyde dehydrogenase 2, ARIC—Atherosclerosis Risk in Communities, ALDH2—Aldehyde dehydrogenase 2, CABG—coronary artery bypass graft, CFAE—complex fractionated atrial electrogram, CHS—Cardiovascular Health Study, CT-computer tomography, CVRF—cardiovascular risk factors, Cx43—connexin43, CYP2E1—Cytochrome P-450 2E1, DTNPV1—deep terminal negativity of P wave in V1, EC—electronic cigarettes, ECG—electrocardiogram, EF—ejection fraction, FASTK—Fas-activated serine/threonine kinase, FGF21—Fibroblast growth factor 21, hEtG—ethyl glucuronide in hair, HRV—heart rate variability, LA—left atrium, LGE—late gadolinium enhancement, hsCRP—high-sensitivity C-reactive protein, hs-cTnT-high-sensitivity cardiac troponin T, hsTNI—high-sensitivity Troponin I, MESA—Multi-Ethnic Study of Atherosclerosis, miR—micro RNA, MRI—magnetic resonance imaging, mRNA—messenger RNA, miRNA—microRNA, MR-proANP—mid-regional fragment of pro atrial natriuretic peptide, NF-κB—nuclear factor-kappa B, NOX—NADPH oxidase, NOX2/gp91phox—NOX2/glycoprotein 91phox, NT-proBNP—N-terminal pro B-type natriuretic peptide, PALS—peak atrial longitudinal strain, PKCε—protein kinase C epsilon, PVI—pulmonary vein isolation, RAAS—renin-angiotensin-aldosterone-system, ROS—reactive oxygen species, SCN5A—voltage-gated Na^+^ channels, TGF-β1—transforming growth factor-β1, TGF-βRII—TGF-β receptor type II, TNF—tumor necrosis factor, TNFR1—TNF-α receptor 1, 4-HNE—4-hydroxy-trans-2-nonenal.
